# A Brainnetome Atlas Based Mild Cognitive Impairment Identification Using Hurst Exponent

**DOI:** 10.3389/fnagi.2018.00103

**Published:** 2018-04-10

**Authors:** Zhuqing Long, Bin Jing, Ru Guo, Bo Li, Feiyi Cui, Tingting Wang, Hongwen Chen

**Affiliations:** ^1^Medical Apparatus and Equipment Deployment, Nanfang Hospital, Southern Medical University, Guangzhou, China; ^2^School of Biomedical Engineering, Capital Medical University, Beijing, China; ^3^Department of Tuberculosis, Beijing Chest Hospital Capital Medical University, Beijing, China; ^4^Department of Traditional Chinese Medicine, Beijing Chest Hospital, Capital Medical University, Beijing Tuberculosis and Thoracic Tumor Research Institute, Beijing, China

**Keywords:** mild cognitive impairment, range scaled analysis, Hurst exponent, brainnetome atlas, support vector machine

## Abstract

Mild cognitive impairment (MCI), which generally represents the transition state between normal aging and the early changes related to Alzheimer’s disease (AD), has drawn increasing attention from neuroscientists due that efficient AD treatments need early initiation ahead of irreversible brain tissue damage. Thus effective MCI identification methods are desperately needed, which may be of great importance for the clinical intervention of AD. In this article, the range scaled analysis, which could effectively detect the temporal complexity of a time series, was utilized to calculate the Hurst exponent (HE) of functional magnetic resonance imaging (fMRI) data at a voxel level from 64 MCI patients and 60 healthy controls (HCs). Then the average HE values of each region of interest (ROI) in brainnetome atlas were extracted and compared between MCI and HC. At last, the abnormal average HE values were adopted as the classification features for a proposed support vector machine (SVM) based identification algorithm, and the classification performance was estimated with leave-one-out cross-validation (LOOCV). Our results indicated 83.1% accuracy, 82.8% sensitivity and 83.3% specificity, and an area under curve of 0.88, suggesting that the HE index could serve as an effective feature for the MCI identification. Furthermore, the abnormal HE brain regions in MCI were predominately involved in left middle frontal gyrus, right hippocampus, bilateral parahippocampal gyrus, bilateral amygdala, left cingulate gyrus, left insular gyrus, left fusiform gyrus, left superior parietal gyrus, left orbital gyrus and left basal ganglia.

## Introduction

Mild cognitive impairment (MCI), which is characterized by memory complaints, attention deficits and other reduced cognitive functions (Petersen, 2007; Han et al., 2011; Zhang et al., 2012), generally represents the transition state between normal aging and the early changes related to Alzheimer’s disease (AD; Desikan et al., 2009; Wang et al., 2015). Overall, MCI patients progress to AD at a rate of 10%–15% per year (Khazaee et al., 2016), and roughly half of them will evolve to AD within 3–5 years (Long et al., 2016). Recently, a great deal of attention from neuroscientists, neurologists and neuroradiologists has been paid to MCI due that efficient AD treatments need early initiation ahead of irreversible brain tissue damages (Davatzikos et al., 2008). Therefore, developing accurate and effective MCI identification methodologies that may be of great importance for clinical interventions of AD are desperately needed.

Functional magnetic resonance imaging (fMRI) has received increasing interests because it could provide a primary method of mechanism detection, diagnostic evaluation or therapeutic monitoring for MCI and AD (Fornito and Bullmore, 2010; Wang et al., 2015). Previous studies demonstrated that the aberrant and spontaneous neuronal activities in MCI or AD could be detected by resting-state fMRI (rs-fMRI; Zhang et al., 2012; Brier et al., 2014), and the abnormal brain regions mainly involved in hippocampus, parahippocampal gyrus, posterior cingulate gyrus and precuneus cortex, etc (Baron et al., 2001; He et al., 2007). In addition, many recent studies employed rs-fMRI data to identify MCI or AD from healthy controls (HCs) by extracting a single type of feature or multi-level characteristics (Chen et al., 2011; Dai et al., 2012; Zhang et al., 2012; Brier et al., 2014; Long et al., 2016), and the recognition accuracies were varied with a wide range, suggesting the MCI or AD discrimination needs to be continued. Generally, an effective rs-fMRI based MCI or AD discrimination method should: (I) exhibit an excellent discrimination accuracy between MCI or AD and HC; (II) specifically quantify fundamental characteristics of Alzheimer’s pathology in individuals with MCI or AD.

Prior studies demonstrated that blood oxygen level dependent (BOLD) signals have been shown scale-free dynamics (Ciuciu et al., 2012; Wei et al., 2013), and the power spectrum of fMRI signals can be written as *S*(*f*) ∝ 1/|*f*|^β^ with *β* < 1 (where *f* represents frequency; Maxim et al., 2005; Gentili et al., 2015), suggesting that the fMRI signals have fractal or fractal-like properties. Hurst Exponent (HE), which has a direct linear relationship with the parameter *β* = 2*HE* − 1, could well display the fractal dynamics of fMRI signals via describing the self-similarity of a time series. In fact, the HE, an index ranging from 0 to 1, could divide the time series into three categories according to its values. A HE bigger than 0.5 indicates a persistent or positively correlated time series, meaning that the time series generally causes changes that fluctuate in the same direction along time. A HE equal to 0.5 stands for a random white noise. A HE smaller than 0.5 implies an anti-correlated or anti-persistent time series. In this case, the dynamics of a time series would keep a reversing pattern in time, and a decrease in the time series generally would be followed an increase and vice versa (Gentili et al., 2015). Recently, HE index has been utilized to measure the changes of BOLD signals related to major depressive disorder, normal and pathological aging, cholinergic modulation, AD, autism disorder and different personality traits (Maxim et al., 2005; Wink et al., 2006; Lai et al., 2010; Lei et al., 2013; Gentili et al., 2015; Jing et al., 2017). However, little information was known about the HE changes in MCI patients, and it still remains unknown whether the HE index could serve as an effective parameter for MCI classification.

In this article, the HE index of fMRI signals were first calculated using range scaled analysis at a voxel level. Then the average HE values of each region of interest (ROI) in brainnetome atlas, a newly structural and functional brain partition scheme, were extracted and compared between MCI and HC groups. At last, the abnormal HE values were adopted as the classification features for a proposed support vector machine (SVM)-based classification method to identify MCI patients from HC.

## Materials and Methods

### Participants

Sixty-nine MCI patients and 63 HC subjects participated in the current study, and none of MCI patients had taken any medications that interfere with cognitive functions. All MCI patients were recruited from the memory outpatient clinic at Nanfang Hospital, and the clinical diagnosis of MCI was made by two experienced neurologists based on the following criteria: (1) memory complaints, confirmed by patient-self or their relatives; (2) normal or near normal performance on cognitive function; (3) normal or near normal activities of daily life; (4) Clinical Dementia Rate equals to 0.5; and (5) absence of dementia according to the DSM-IV (Diagnostic and Statistical Manual of Mental Disorders, 4th edition, revised). The HC participants matched well with MCI patients on gender, age and education level and were collected from local community by print advertisements, and the inclusions for all participants were: (1) no other nervous or psychiatric diseases that can intervene with cognitive functions, such as Parkinson’s disease, depressive disorders and encephalitis, etc; (2) no history of stroke or dependence of alcohol; (3) no systemic diseases that cause cognitive impairments; and (4) no medication conditions that can influence cognitive performance. All subjects were undergone several clinical assessments including Clinical Dementia Rate, Mini-Mental State Examination (MMSE) and Auditory Verbal Learning Test (AVLT). This study was approved by the ethics committee of Nanfang Hospital affiliated to Southern Medical University, and the informed written consents from all subjects were obtained in accordance with the Declaration of Helsinki. Five MCI patients and three HC subjects were discarded due to excessive head motion during the scan, and the detailed clinical characteristics of the remaining participants were summarized in Table 1.

**Table 1:** Participants’ demographic and clinical characteristics.

Characteristics	MCI	HC	*P* values
Gender (M/F)	64 (28/36)	60 (26/34)	0.96^#^
Age (years)	67.14 ± 7.33	65.27 ± 7.30	0.16^*^
Education (years)	9.73 ± 4.24	10.07 ± 4.27	0.66^*^
CDR	0.5	0	0^*^
MMSE	23.16 ± 2.77	27.40 ± 3.15	<0.001^*^
AVLT-immediate recall	7.99 ± 2.60	12.94 ± 2.94	<0.001^*^
AVLT-delay recall	3.64 ± 2.89	9.77 ± 2.79	<0.001^*^
AVLT-recognition	7.11 ± 3.55	11.58 ± 2.23	<0.001^*^

Values are mean ± SD unless the SD was not calculated. M, male; F, female. CDR, Clinical Dementia Rating scale; MMSE, Mini-Mental State Examination; AVLT, Auditory Verbal Learning Test. ^#^The P value was obtained by Chi-square test. ^*^The P values were obtained by two-sample two-tailed-t-test.

### Data Acquisition

All images were collected on a 3 Tesla Siemens scanner with 8-channel radio frequency coil at Nanfang hospital. Headphones and a foam padding were utilized to reduce the scanner noise and limit the head motion during the scan, and all subjects were instructed to close their eyes, to keep mind relax, to not fall asleep and to not move their head. Resting-state fMRI were acquired using an echo-planar imaging sequence with the following parameters: repetition time = 2000 ms, echo time = 40 ms, flip angle = 90°, matrix size = 64 × 64, number of slices = 28, field of view = 240 × 240 mm^2^, slice thickness = 4 mm, and voxel size = 3.75 × 3.75 × 4 mm^3^. Two-hundred and thirty-nine volumes were collected for each subject within 478 s. T1-weighted structural images for all subjects were acquired by using magnetization-prepared rapid gradient echo sequence with the following parameters: repetition time = 1900 ms, echo time = 2.2 ms, inverse time = 900 ms, flip angle = 9°, matrix = 256 × 256, number of slices = 176, slice thickness = 1 mm, and voxel size = 1 × 1 × 1 mm^3^.

### Data Preprocessing

Data preprocessing for all images were carried out with Statistical Parametric Mapping (SPM8)^1^. The first 10 functional volumes were discarded due to signal equilibrium and participant’s adaptation to the scanner environment, and the remaining 229 volumes were corrected for different acquisition time between slices. Then all volumes were realigned to the first volume by using a six-parameters rigid-body spatial transformation to compensate for head movement effects. Eight participants (five MCI patients and three HC subjects) were discarded because of excessive head motion (2 mm and 2°criteria). To improve the spatial normalization accuracy, the realigned images were normalized into the Montreal Neurological Institute space by using the parameters obtained from structural normalization, and all normalized functional images were re-sampled into a voxel size of 3 × 3 × 3 mm^3^. Next, all the normalized images were detrended, and the spurious covariates including the six head motion parameters obtained from rigid-body transformation, signals of white matter and ventricular system were regressed. At last, a temporal band-pass filter (0.01–0.10 HZ) was carried out on the time series of each voxel to reduce the effects of low-frequency drifts and high-frequency cardiac and respiratory noise, and the filtered images were smoothed with a 4 mm full width at half maximum Gaussian kernel.

### HE Calculation and Feature Selection

**Figure 1:**
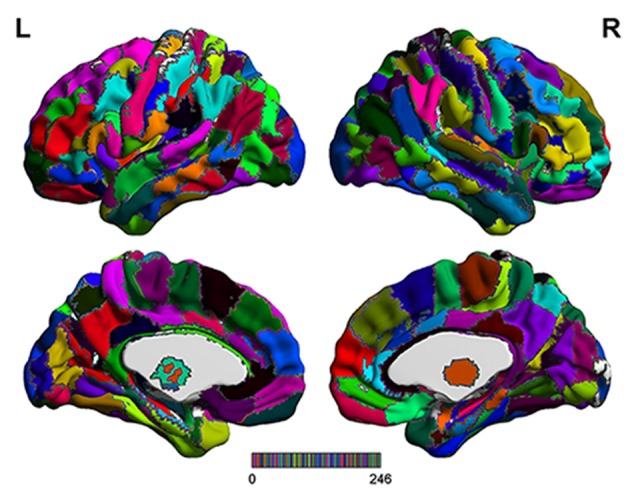
The detailed brainnetome atlas which including 210 cortical sub-regions and 36 subcortical sub-regions.

The range scaled analysis, which is an effective method to detect the temporal complexity of a time series, was utilized to calculate the HE index of fMRI signals at a voxel level, and the detailed principle of HE calculation was reported in our previous study (Jing et al., 2017). In addition, the brainnetome atlas (Figure 1), which partitions the cerebral cortex into 246 ROIs including 210 cortical sub-regions and 36 subcortical sub-regions (Fan et al., 2016), was used to extract the HE index feature for the SVM-based classification algorithm. In this article, the average HE values of each ROI in brainnetome atlas were extracted as the candidate features. Considering that properly and correctly reducing the number of features could not only improve the classification performance but also speed up the computation (De Martino et al., 2008; Pereira et al., 2009). Thus a Fisher score method and two-sample two-tailed-*t*-test (*P* < 0.05, uncorrected) were utilized to select out the discriminative HE features between MCI patients and HC subjects. The detailed Fisher score criterion for each candidate feature is defined as:

(1) $$FS\;=\;\frac{n_{1}{\left(m_{1}-m\right)}^{2}+n_{2}{\left(m_{2}-m\right)}^{2}}{n_{1}\sigma_{1}^{2}+n_{2}\sigma_{2}^{2}}$$

Here *n*_1_ and *n*_2_ are the number of the samples on each group, *m*_1_ and *m*_2_ are the respective mean value of the feature, *m* represents the mean value of the feature, $\sigma_{1}^{2}$ and $\sigma_{2}^{2}$ represent the variance of the feature on each group. A high Fisher score value indicates a strong discriminative ability of the feature to some degree. At last, it’s worth noting that the feature selection was only performed on the training set of per leave-one-out cross-validation (LOOCV) fold, which could reduce the overfitting of the classification algorithm.

### SVM-Based Classification Method

The SVM algorithm, which has been widely utilized for its powerful recognition function as well as its simple theory and implementation, was originally proposed for binary classification problems based on statistical learning principles (Beheshti and Demirel, 2016). During the training process, the SVM algorithm seeks the optimal separation hyper-plane in the feature space where the input features were mapped into using a kernel function, and each divided subspace corresponds to one class of training set. In the same way, all the test samples could be labeled depending on which subspace they are mapped into after the training process (Magnin et al., 2009). In this article, the LibSVM toolbox^2^ was utilized for SVM implementation.

**Figure 2:**
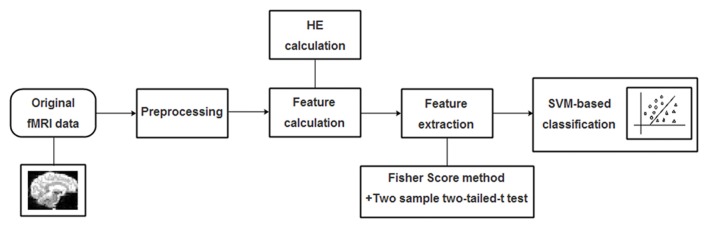
A flowchart of the proposed support vector machine (SVM)-based classification method for mild cognitive impairment (MCI) identification.

The radial basis function (RBF) defined as (*X,X*_i_) → *K(X,X*_i_) = *e*^*γ*|*X*−*X*_i_|^2^^ was adopted as the kernel function for the SVM algorithm. To improve classification performance, a grid-search method was utilized to optimize two parameters: the parameter *γ* representing the width of RBF kernel and the punishment factor *C* adjusting the importance of error separation. In detail, at each pair of (*γ*, *C*), three steps including the above-mentioned feature selection, the training of the SVM-based algorithm and the prediction of the test samples were performed in succession, and the classification performance was estimated with LOOCV. It’s worth noting that the feature selection was only carried out on the training set of each LOOCV fold. The whole classification process was repeatedly performed with (*γ*, *C*) varying along a grid with *γ* = 2^−8^, 2^−7.5^,…,2^8^ and *C* = 2^−8^, 2^−7.5^,…,2^8^, which is referred as the grid-search method. Considering that each pair of (*γ*, *C*) corresponds to an accuracy, the best accuracy rate on the grid of 33 × 33 was acquired as the classification accuracy of the classifier. A flowchart of the detailed classification process was shown in Figure 2.

## Results

**Figure 3:**
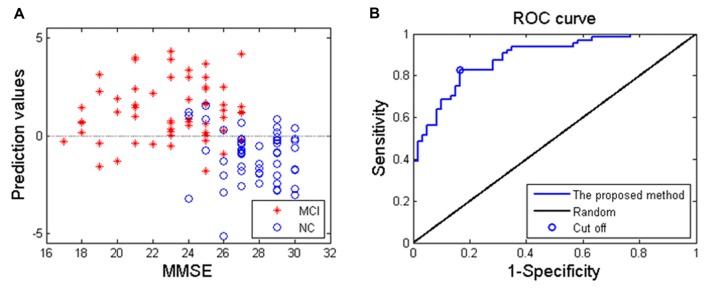
**(A)** The relationship between the Mini-Mental State Examination (MMSE) score of MCI patients and prediction values; **(B)** receiver operating characteristics curve of the proposed classification method, and the area under curve is 0.88.

Applying the proposed SVM-based classification method to identify MCI patients from HC subjects, our results indicated 83.1% accuracy, 82.8% sensitivity and 83.3% specificity. Besides, the receiver operating characteristics curve and the relationship between MMSE and prediction values were shown in Figure 3, and the area under curve of the classification algorithm is 0.88, indicating a powerful classification performance.

**Figure 4:**
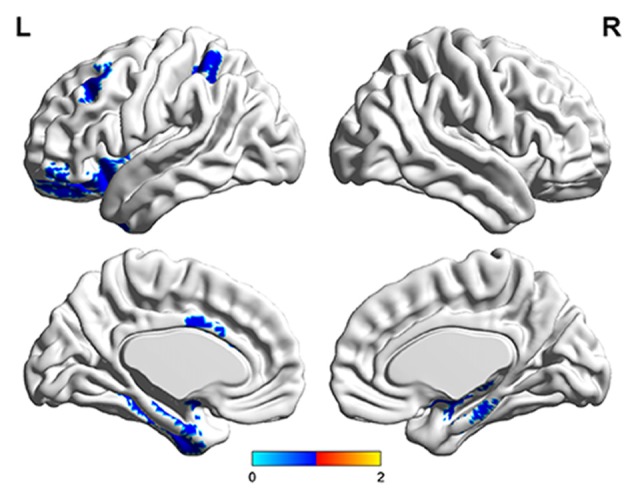
The brain regions with abnormal hurst exponent (HE) values in MCI patients by using brainnetome atlas.

**Figure 5:**
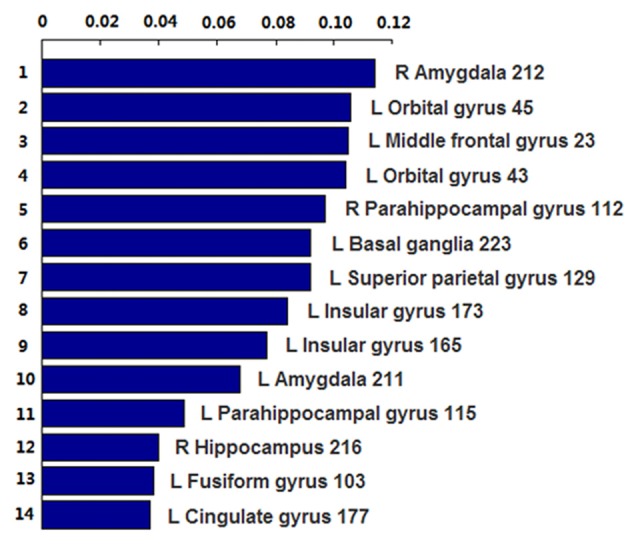
The Fisher score values of these abnormal HE features which were retained no less than 118 times (124 × 0.95, 124 is the total number of the samples) in the whole leave-one-out cross-validation (LOOCV) process.

The number of features retained in per fold of LOOCV was shown in Table 2. In addition, the abnormal HE brain regions with the retained times of the HE features no less than 118 (124 × 0.95, 124 is the total number of the samples) in the whole LOOCV process were shown in Figure 4, and the Fisher score values of these abnormal HE features were displayed in Figure 5. Compared to HC subjects, these abnormal HE brain regions in MCI patients were predominately involved in left middle frontal gyrus, right hippocampus, bilateral parahippocampal gyrus, bilateral amygdala, left cingulate gyrus, left insular gyrus, left fusiform gyrus, left superior parietal gyrus, left orbital gyrus and left basal ganglia.

**Table 2:** The number of features retained in per fold of leave-one-out cross-validation (LOOCV) with brainnetome atlas.

Fold	No. of features	Fold	No. of features	Fold	No. of features	Fold	No. of features
1	16	32	17	63	15	94	15
2	15	33	16	64	16	95	15
3	17	34	16	65	15	96	15
4	17	35	14	66	14	97	15
5	16	36	15	67	16	98	15
6	16	37	14	68	16	99	15
7	16	38	15	69	15	100	16
8	15	39	15	70	16	101	14
9	15	40	16	71	15	102	15
10	15	41	16	72	16	103	16
11	15	42	15	73	14	104	14
12	15	43	14	74	15	105	15
13	14	44	14	75	14	106	14
14	16	45	16	76	16	107	15
15	16	46	16	77	16	108	16
16	15	47	16	78	17	109	15
17	14	48	15	79	16	110	16
18	15	49	14	80	16	111	16
19	15	50	17	81	16	112	15
20	15	51	15	82	16	113	15
21	16	52	14	83	15	114	17
22	15	53	14	84	14	115	15
23	16	54	14	85	16	116	16
24	15	55	14	86	16	117	14
25	17	56	15	87	16	118	14
26	15	57	16	88	14	119	15
27	16	58	16	89	16	120	15
28	16	59	15	90	14	121	16
29	16	60	16	91	16	122	15
30	14	61	16	92	16	123	15
31	14	62	14	93	15	124	14

## Discussion

This study proposed an effective classification method to identify MCI patients from HC subjects using HE index of rs-fMRI. A promising classification performance was obtained with an accuracy of 83.1% and an area under curve value of 0.88, suggesting that the proposed SVM-based method was effective in identifying MCI from HC subjects, and the calculated HE index could serve as an effective feature for the SVM-based classification algorithm.

To obtain high discrimination accuracy for MCI classification, three steps were taken for the proposed classification method. First, previous studies demonstrated that properly reducing the number of features could not only improve the classification performance but also speed up the computation (Dosenbach et al., 2010; Dai et al., 2012). Thus two-sample two-tailed-*t*-test and Fisher score criteria were both utilized to select out the discriminative HE features in this article, and the classification performance was improved significantly compared to without feature selection. In fact, we firstly tried a total 246 HE features by using the proposed SVM-based algorithm, and the classification accuracy without feature selection was lower than 70%. It needs to note that the feature selection was only performed on the training set, which could reduce the overfitting of the classifier. Second, the RBF kernel function was adopted as the kernel function due that it could deal with the case when the relationship between labels and features is nonlinear (Hsu et al., 2003), which also has an important impact on classification performance. In this article, we also utilized the linear kernel function for MCI classification, and the discrimination rate was 78.2%, which was lower than that with RBF kernel. At last, the grid search method, which has a high learning accuracy and could be implemented with parallel processing (Long et al., 2016), was utilized to optimize the two parameters of SVM, which also improved the classification performance. In addition, to further validate the effectiveness of the proposed MCI classification method, the dataset was randomly split into two subsets including a training subset (42 MCI and 40 HC), a testing subset (22 MCI and 20 HC). The training subset was utilized to train the classification algorithm and optimize the two parameters through an internal cross-validation procedure which averagely divided the training set into two groups to train the algorithm with one group and then predict the other group mutually (Dyrba et al., 2015). Then the final performance of the classification algorithm was estimated with the testing subset. A promising accuracy of 85.71% was obtained, which also indicated that the proposed SVM-based method is effective in identifying MCI patients form HC subjects.

In this article, we found that the abnormal HE brain regions in MCI patients mainly involved in left middle frontal gyrus, right hippocampus, bilateral parahippocampal gyrus, bilateral amygdala, left cingulate gyrus, left insular gyrus, left fusiform gyrus, left superior parietal gyrus, left orbital gyrus and left basal ganglia. Almost all these brain regions were consistent with previous studies that analyzed the structural and functional data of MCI or AD patients with conventional statistical analysis (Hirata et al., 2005; Lerch et al., 2008; Xie et al., 2012). The middle frontal gyrus, hippocampus, parahippocampal gyrus, cingulate gyrus and orbital gyrus belong to the default mode network (Dai et al., 2012; De Vogelaere et al., 2012). Currently, the behavioral correlations of default mode network still remain uncharacterized although some investigators had proposed several potentially inclusive hypotheses that it mediates processes such as reviewing past knowledge and preparing for future actions (Greicius et al., 2004). The abnormal HE values in these brain regions supplementarily supported the abnormalities of default mode network in MCI patients. In addition, the amygdala and insular gyrus were labeled with significant atrophy in MCI patients in previous voxel-based morphometry studies (Hämäläinen et al., 2007), and the fusiform gyrus showed significantly aberrant amplitude of low-frequency fluctuations of BOLD signals in MCI (Wang et al., 2015). Furthermore, the basal ganglia was associated with cognitive functions such as mood swings or disorders (de Oliveira and de Oliveira, 2013). All the above-mentioned evidences suggested that these abnormal brain regions were related to the mechanisms underlying MCI patients.

The HE analysis has already been utilized to describe complex properties of biological signals including electroencephalogram and electrocardiogram (Costa and McCrae, 1992; Ignaccolo et al., 2010). By applying the HE analysis method to BOLD signals, some investigators found that the HE value of fMRI signals in gray matter was higher than in white gray (Maxim et al., 2005), and decreased with cholinergic transmission enhancement and augmented in hippocampus with aging (Wink et al., 2006). Nevertheless, these findings could not conclude that a higher HE value is associated with worse brain functioning. It seems to reflect some inherent patterns of spontaneous discharge and the HE could be modulated by different psychotic or psychological variables (Gentili et al., 2015). In this article, the HE analysis was applied in MCI patients, and some core brain regions were detected with HE abnormalities. It demonstrated that the persistent behavior of brain activities in these abnormal regions were changed, which may provide some information for the mechanisms underlying MCI patients. However, the physiological significance of HE index still remains unknown currently, and future studies should pay more attention to confirm it through the multi-modal imaging validation in animal models.

Several issues need to be addressed in this article. First, some other structural or functional brain partition atlases exist and these brain parcellation atlases could also be used for identifying different psychiatric disorders. Different parcellation schemes may lead to different classification results. Compared to the widely used automated anatomical labeling atlas, the brainnetome atlas that simultaneously combines information from structural and functional connections obtained better classification performance in differentiating major depressive disorder from HC in our previous study (Jing et al., 2017). Thus the brainnetome atlas was adopted to discriminate MCI from HC subjects in this work. Second, deep learning plays an increasing important role in identifying different psychiatric disorders as it could acquire powerful identification performance from high dimension feature data. Future studies could extract the HE features or other multi-level characteristics at a voxel level for deep learning algorithm to obtain better classification accuracy and more comprehensive explanations for abnormalities in psychiatric disorders.

## Author Contributions

ZL, BJ and RG made substantial contributions to the conception, design, analysis and interpretation of data and drafted the manuscript. ZL, BJ, BL and HC made contributions to the revision of the manuscript. ZL, FC and TW made contributions to the data acquisition. HC, the corresponding author, made contributions to conception and interpretation of data, and determined the final version to be submitted for publishing. All authors read and approved the final manuscript.

## Conflict of Interest Statement

The authors declare that the research was conducted in the absence of any commercial or financial relationships that could be construed as a potential conflict of interest.

## Footnotes

^1^http://www.fil.ion.ucl.ac.uk/spm

^2^http://www.csie.ntu.edu.tw/~cjlin/libsvm

## Abbreviations

AD: Alzheimer’s disease
BOLD: blood oxygen level dependent
fMRI: functional magnetic resonance imaging
HCs: healthy controls
HE: Hurst exponent
LOOCV: leave-one-out cross-validation
MCI: mild cognitive impairment
RBF: radial basis function
ROI: region of interest
rs-fMRI: resting-state functional magnetic resonance imaging
SVM: support vector machine.
